# Design of an Intramuscular Injection Simulator: Accommodating Cultural Differences

**DOI:** 10.7759/cureus.18980

**Published:** 2021-10-22

**Authors:** Julia Micallef, Andrea C Lin, Artur Arutiunian, Adam Dubrowski

**Affiliations:** 1 Health Sciences, Ontario Tech University, Oshawa, CAN; 2 Internal Medicine/Hospital-Based Medicine, Singapore General Hospital, Bukit Merah, SGP

**Keywords:** medical education, cultural context, singapore, simulation, intramuscular injection

## Abstract

We had developed an inexpensive intramuscular (IM) injection simulator and gathered feedback from Canadian hospital-based practicing nurses about the design features of the simulator. While the feedback critiqued the density of the simulator as being too stiff and suggested making the shape more realistic, it was also unanimously agreed that this IM injection simulator is more realistic than any other previous models they have used, therefore deeming it an acceptable training tool for nursing students in Canada. For this simulator to serve as a training tool in other countries, such as Singapore, we partnered with SingHealth, a hospital network in Singapore, to conduct identical product testing in a different ethnic context and compare the data to our previous work. This article is based on this study. We had 21 nurses from Singapore General Hospital test the IM injection simulator and fill out the same survey the Canadian nurses had done. With a 100% response rate, only 26% of the Singapore hospital-based nurses agreed that this IM injection simulator is a more ethnically appropriate representation of anatomy than previous simulators they have used. There were numerous other differences in feedback compared to the Canadian nurses, such as the fat layer being too thick. These differences in feedback highlight the importance of including ethnicity as a factor during the design of simulators. Therefore, despite the silicone IM injection simulator being a cost-effective solution to practice IM injections, the features of the simulator need to be improved to make it a valuable teaching tool for nursing students, especially those in Singapore.

## Introduction

Intramuscular (IM) injections are a common route of administration for medicine in clinical settings. Nursing students need a lot of drill-like, repetitive practice to perform this invasive skill without harm to patients [1]. Currently, there are two types of simulators being used for training: animal and synthetics [2]. Animal models are realistic, but there are issues with the ethics of using these: standardization, health, and safety. On the contrary, synthetic models are good alternatives; however, they are costly, often lack proper representations of anatomical features, and do not provide the variability that is often needed for learning [3]. With the advent of additive manufacturing, such as 3D printing, we have developed and tested inexpensive IM injection simulators directed to nursing students that allow aspiring nurses to become well acquainted with the skill before entering the clinical setting [2].

Our previous report [2] focused on gathering feedback from Canadian hospital-based practicing nurses about the design features of the simulator. In general, the feedback was positive and helpful for additional improvements. The main areas of concern voiced by the Canadian nurses were the densities of the layers being too stiff such that the experience does not optimally simulate performing the IM injection on a patient. Additionally, the nurses noted that the shape of the simulator should be molded to be closer to that of the human body for a more realistic simulation. However, despite the critiques, all the volunteer nurses unanimously agreed that this new IM injection simulator provides a better representation of anatomical features than any other previous simulator they have used. Therefore, based on the feedback, we concluded that the simulator was an acceptable and feasible solution for rural and remote areas.

To be accepted internationally, and more specifically in Singapore, as a training tool, we wanted to explore the perceptions of Singaporean nurses of the ethnically appropriate representations of anatomy represented by the simulator. We partnered with SingHealth, a hospital network in Singapore, to conduct identical product testing in a different ethnic context. Our recent collaboration with a hospital training system in Singapore, where the Singaporean nurses used the IM injection simulator for training, highlighted some important educational challenges in using the simulator developed by Canadians in a Singaporean context.

The main purpose of this report was to compare the data from our previous report (with Canadian nurses) to the data we received from nurses who train and practice nursing in Singapore to help inform our redesigning of the initial IM injection simulator to fit a Singaporean context.

## Materials and methods

**Figure 1:**
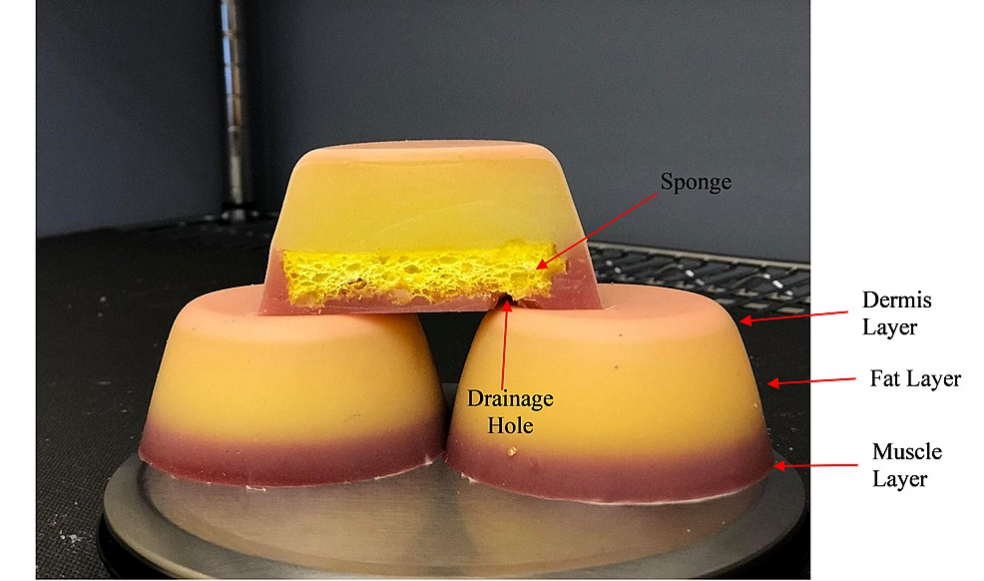
Side and cross-sectional view of IM simulators.

Originally, as described in the previous report [2], the IM injection simulators, shown in Figure 1, were designed to be used as a training tool for pre-licensure nursing students and to train first responders. As a low-cost simulator, it may also have a utility for the maintenance of skills for in-hospital nursing staff and in rural and remote areas. However, for this report, we were interested in assessing the design features, and elements of acceptability and feasibility in the use of this simulator in training in-hospital nursing staff in an international context. Specifically, we were interested in using it with the Singaporean context as the patient population is significantly different, with the main differences being related to the body size, body composition, and adipose tissue distribution, when compared to the North American population [4].

The inputs necessary for the design and fabrication of the simulators were described in the previous report [2]. The participants were 21 nursing educators and nursing clinical instructors from the Institute of Advanced Nursing (IAN), Singapore General Hospital (SGH) who are involved in in-house training for students and new hires in the hospital. They conduct a clinical assessment on students and new hires on various nursing skills including performing IM injections. The participants had a minimum of eight years of experience in the clinical area. The development team contacted the IAN at SGH to ask the participating nurses to try the IM injection simulators. All participants were informed that the development of this simulator is not related to their work and professional development activities and that their participation is voluntary and anonymous. They were also told that they would be sent an anonymous Google Forms link with a survey to provide feedback on the simulator. The participants did not know the development team, and none of the members of the development team knew any of the participants. We asked the participants to perform IM injections on the new simulator and provide us with feedback on possible improvements. 

In this report, we provided the nurses from IAN identical IM injection simulators, each paired with a one-inch (25.4 mm) long, 22-gauge needle, an alcohol wipe, and water to act as the injecting liquid. Each participant performed the injection a minimum of three times using the simulator and the provided tools. After that, they were sent a link to a survey via Google Forms to provide us feedback that assessed the representations of anatomical features of the IM injection pad, how it compared to previous simulators, and possible improvements to the simulator. The survey was based on a prior publication in which the authors tested the efficacy of their developed silicone simulator for perineal repair suturing [5]. There was a total of 18 questions that included comparing the effectiveness and correctness of the anatomical features of previous simulators to the newly developed simulator, questions assessing whether they would recommend this simulator as an educational tool for nursing students, rating on a scale of 1-10, the realism, for this survey operationally defined as the appropriateness of the anatomical features, of various components of the simulator, and suggestions for further improvements (Table 1). We had a total of 21 volunteer nurses fill out the survey anonymously, giving us a 100% response rate.

**Table 1:** List of survey questions provided to the nursing educators and nursing clinical instructors from the lAN, Singapore General Hospital. IAN: Institute of Advanced Nursing

Question Number	Question
1	What is an intramuscular injection simulation that you have used in the past, if you have used any?
2	If you have previously used another intramuscular injection simulation, how effective was it in increasing your competency on the procedure?
3	If you have previously used another intramuscular injection simulation, how effective was it in increasing your confidence in performing the procedure?
4	If you have used other intramuscular injection simulations before, how does this injection pad compare to the previous simulator?
5	Please elaborate on your answer to question 4.
6	Would you recommend the use of the new injection pad to assist with training and education of intramuscular injections for students?
7	To serve as a practice simulation of an intramuscular injection, please rate how realistic the colour of the injection pad is.
8	To serve as a practice simulation of an intramuscular injection, please rate how realistic the thickness of the Dermis layer of the injection pad is.
9	To serve as a practice simulation of an intramuscular injection, please rate how realistic the softness of the Dermis layer of the injection pad is.
10	To serve as a practice simulation of an intramuscular injection, please rate how realistic the softness of the Fat layer of the injection pad is.
11	To serve as a practice simulation of an intramuscular injection, please rate how realistic the softness of the Muscle layer of the injection pad is.
12	How would you improve the new injection pad?
13	Did you experience any difficulties or discomfort while using the simulator?
14	Can you feel the difference between the different layers (dermis, fat, and muscle)? If not, is it important that there is a definite distinction, or is it good as is?
15	Was the sponge effective in absorbing the liquid?
16	Was the drainage hole (found on the bottom of the injection pad) effective in draining the liquid from the injection pad?
17	Do you have any additional comments you would like to note?
18	How often do you perform intramuscular injections in clinical settings?

## Results

The survey data were considered ordinal data, although the debate is open whether this type of data can be interpreted using parametric or non-parametric statistics [6,7]. Since our participant numbers are low and because the point of the analysis was to inform the design, rather than to provide evidence of validity, we decided not to use inferential statistics but instead present the data in the form of descriptive statistics. As such, data are presented both as frequencies of distribution as per each question as well as mean and standard deviations. These dual representations of data enable a more informed discussion that leads to informing our designs, or more specifically, the redesign needs in the Southeast Asian context. All quantitative data are presented in Table 2 and Table 3.

**Table 2:** Response frequency of questions 2-4 and 6 from the survey given to the nursing educators and nursing clinical instructors from the lAN at SGH. IAN: Institute of Advanced Nursing; SGH: Singapore General Hospital

Question Number	Not at all effective	Slightly effective	Neutral	Effective	Very effective	Total responses
2	0	3	11	6	1	21
3	0	2	10	8	1	21
	Less Realistic	Neutral	More Realistic			
4	6	8	7			21
	Strongly Disagree	Disagree	Neutral	Agree	Strongly Agree	
6	1	7	4	7	2	21

**Table 3:** Response frequency of questions 7-11 from the survey given to the nursing educators and nursing clinical instructors from the lAN at SGH to rate the realism of the IM injection on a scale of 1 (not realistic) to 10 (very realistic). IAN: Institute of Advanced Nursing; SGH: Singapore General Hospital

Question Number	Frequency Scale From 1 (least realistic) to 10 (most realistic)										Total Number of Responses	Average Response	Standard Deviation
	1	2	3	4	5	6	7	8	9	10			
7	0	0	0	2	4	3	6	3	3	0	21	6.62	1.53
8	1	0	2	5	3	3	4	2	1	0	21	5.38	1.94
9	1	0	5	4	3	1	2	3	2	0	21	5.19	2.26
10	1	0	3	6	2	4	2	2	1	0	21	5.10	1.95
11	1	0	3	7	0	4	4	1	1	0	21	5.10	1.95

Of the 21 nurses that filled out the survey, only seven agreed that the new IM injection simulation was more realistic, defined as ethnically appropriate representations of anatomy, than any previous IM simulator that they have used (Table 2, question 4). The previous simulators used to practice IM injections were noted as stress balls, manikins, and sponges. Interestingly, one of the participants noted in question 5 that “the texture (of the new IM injection simulator} is harder than the usual one used, but more realistic during the insertion phase” when comparing the appropriateness of the anatomical features of the IM injection simulator to previous models used. Additionally, only nine participants agreed that they would recommend the new IM simulator to assist in the training and education of nursing students with 9.5% strongly agreeing and 33.3% agreeing (Table 2, question 6). When it came to ranking the anatomical representations of tissues, the results indicate an overwhelmingly neutral response with questions 8-11 all having an average rating of around five(Table 3). The only component of the IM injection simulator that produced a favorable response was question 7, which asks participants to rate the colors used to make the simulator (Table 3).

The comments that the nurses provided were analyzed and categorized into themes. These include: 1. Difficulties encountered when using the simulator; 2. Areas of improvement.

Regarding the first theme displayed in the comments provided by nursing educators and nursing clinical instructors from lAN via question 13 of the survey, many noted the same difficulties when using the IM injection simulator. One participant notes “the model gives a good simulation for introducing the needle into the skin; however, it was difficult to push fluid into the model.” This was one of the many comments that noted the resistance and pressure encountered when trying to inject liquid into the IM injection simulator. Another noteworthy comment states “the liquid injected seems to rebound into the syringe after administration,” which aligns with the other comments that note the injecting liquid would “splash out (of the IM injection simulator} when removing the needle.”

When asked about how to improve the IM injection simulators in question 12 of the survey, there once again was a consensus as to what should be done. Numerous comments were suggested to make the dermis, fat, and muscle layers softer, with one participant commenting “suggest to have a softer texture, similar to real human flesh.” A very interesting suggestion was “to have the fat layer thinner so that participants can reach the muscle layer without pushing the needle in.” The idea of making the fat layer thinner was a common occurrence, with another participant suggesting that “the adipose tissue can be of a lesser thickness.” In addition to reducing the thickness of the fat layer, a few participants suggested “reduce(ing) the thickness of dermis (layer}”, as well. Overall, it was noted that the “(IM injection} pad is too thick, making it difficult to perform 'withdrawal of plunger to check for blood' technique.” Another common improvement was regarding the sponge in which one participant noted to add “more sponge inside to absorb more fluid,” and another suggested to “place sponge higher as fluid leakage is noted on the removal of the needle.” Other participants suggested, “to consider removable sponge for cleansing and reusing.” Finally, another common suggestion of improvement was to “change the shape,” to be more realistic as another participant similarly notes that they “prefer the realistic anatomical landmarks manikin.”

## Discussion

The main purpose of this report was to compare the perceived satisfaction with the custom-designed, inexpensive simulator received from Canadian and Singaporean nurses, to help inform our iterative designing of the initial IM injection simulator to fit a Singaporean context. The results revealed numerous differences in responses when comparing the current data set obtained from nurses in Singapore to the earlier report with Canadian in-hospital nurses. Specifically, regarding the overall representation of the anatomical features of the simulator, the nurses in Singapore did not think this IM injection simulator provided better representations of these features compared to other simulators they have used in the past. This was contrary to the response from the nurses in Canada, who unanimously agreed that the IM injection simulator was a better representation compared to previous simulators that they used during training. This is interesting as the previous simulators noted by the nurses from both countries were the same, namely sponges and manikins. Another notable difference in ratings between the two groups of nursing can be seen in the average responses to questions 7-11 in the survey, which asks the participants to rate the various components of the IM injection simulator in terms of their representations of the anatomical features. The average rating by the nurses from Singapore was 5/10 (Table 3), while the average ratings from Canadian nurses were 8/10 [2]. Collectively, these data show that the nurses in Singapore perceived the simulator as a moderate representation of anatomical features and considerably less representative than similar simulators that they used in the past, while their Canadian counterparts perceived the simulator as a good representation, and considerably more representative when compared to other simulators used in training.

The analyses of the qualitative feedback received from the Singaporean nurses, compared to the feedback from the Canadian nurses, provided us with a plausible explanation for the differences. Specifically, all the nurses from SGH critiqued the thickness of the IM injection simulator, with an emphasis on the fat layer being too thick, while the Canadian nurses did not note this as being an issue. That is, the simulator being too thick is what hindered the perceived representations of the anatomical features for the SGH nurses. This brings to question the contextual differences between the patients in Canada and Singapore. It was mentioned by the SGH nurses that the Southeast Asian patients, of which the Singapore patients were a subset of, tend to be small and thin in build, possibly making this IM injection simulator less representative of the patients in Singapore. This aligns with research that indicates that Southeast Asian ethnicities tend to have a lower BMI compared to other ethnicities [8]. However, Southeast Asian ethnicities tend to have a higher abdominal body fat percentage compared to other ethnicities [9]. One of the few studies that included Southeast Asians while investigating race differences in subcutaneous fat across multiple sites (i.e., not only abdomen) showed that Southeast Asians, as compared with Caucasians and African Americans, had significantly smaller leg and gluteus fat mass, and greater trunk fat mass [10]. Given that, globally, nurses learn three possible IM injection sites, which are the arm (deltoid); thigh (vastus lateralis); upper outer posterior buttock (gluteus maximus), and not the abdomen, it is possible that the thickness of the simulated adipose tissue in the current simulator was too thick for the nurses from SGH, while adequate for the Canadian nurses. Based on this feedback, the simulator will need to be redesigned so that each layer is thinner to better represent the patient demographics in Singapore, and more generally for the patient population.

In summary, this report highlights the importance of including ethnicity as a factor during the design of simulators - a factor that, to the best of our knowledge, has not been integrated into the design cycles. Although the simulation industry has made great efforts to increase the ethnic diversity of simulators offered, this diversity is still mainly “skin deep”. With the use of additive manufacturing techniques to customize design and building, our findings suggest that educators who use simulation and developers who may build simulators locally should pay attention to other, anthropometric ethical differences.

## Conclusions

While the silicone IM injection simulator can be a cost-effective solution to practice IM injections, the representations of tissues that the simulators are intended to mimic need to be improved to make it a valuable teaching tool for nursing students. With the feedback from the Singapore nurses, we can now redesign the simulator to better represent the patients in Singapore.
